# Casein kinase 2-mediated phosphorylation of Brahma-related gene 1 controls myoblast proliferation and contributes to SWI/SNF complex composition

**DOI:** 10.1074/jbc.M117.799676

**Published:** 2017-09-22

**Authors:** Teresita Padilla-Benavides, Brian T. Nasipak, Amanda L. Paskavitz, Dominic T. Haokip, Jake M. Schnabl, Jeffrey A. Nickerson, Anthony N. Imbalzano

**Affiliations:** From the ‡ Department of Biochemistry and Molecular Pharmacology, University of Massachusetts Medical School, Worcester, Massachusetts 01605 and; the § Department of Pediatrics, University of Massachusetts Medical School, Worcester, Massachusetts 01655

**Keywords:** cell proliferation, chromatin regulation, chromatin remodeling, phosphorylation, transcription, Brg1, SWI/SNF subunits, casein kinase 2, myoblast

## Abstract

Transcriptional regulation is modulated in part by chromatin-remodeling enzymes that control gene accessibility by altering chromatin compaction or nucleosome positioning. Brahma-related gene 1 (Brg1), a catalytic subunit of the mammalian SWI/SNF chromatin-remodeling enzymes, is required for both myoblast proliferation and differentiation, and the control of Brg1 phosphorylation by calcineurin, PKCβ1, and p38 regulates the transition to differentiation. However, we hypothesized that Brg1 activity might be regulated by additional kinases. Here, we report that Brg1 is also a target of casein kinase 2 (CK2), a serine/threonine kinase, in proliferating myoblasts. We found that CK2 interacts with Brg1, and mutation of putative phosphorylation sites to non-phosphorylatable (Ser to Ala, SA) or phosphomimetic residues (Ser to Glu, SE) reduced Brg1 phosphorylation by CK2. Although BRG1-deleted myoblasts that ectopically express the SA-Brg1 mutant proliferated similarly to the parental cells or cells ectopically expressing wild-type (WT) Brg1, ectopic expression of the SE-Brg1 mutant reduced proliferation and increased cell death, similar to observations from cells lacking Brg1. Moreover, pharmacological inhibition of CK2 increased myoblast proliferation. Furthermore, the *Pax7* promoter, which controls expression of a key transcription factor required for myoblast proliferation, was in an inaccessible chromatin state in the SE-Brg1 mutant, suggesting that hyperphosphorylated Brg1 cannot remodel chromatin. WT-, SA-, and SE-Brg1 exhibited distinct differences in interacting with and affecting expression of the SWI/SNF subunits Baf155 and Baf170 and displayed differential sub-nuclear localization. Our results indicate that CK2-mediated phosphorylation of Brg1 regulates myoblast proliferation and provides insight into one mechanism by which composition of the mammalian SWI/SNF enzyme complex is regulated.

## Introduction

Chromatin-remodeling enzymes are large, multisubunit complexes that hydrolyze ATP to disrupt histone/DNA contacts and modify chromatin structure by increasing or decreasing its accessibility to regulatory factors controlling transcription, replication, recombination, and repair (1–3). The mammalian SWI/SNF complexes are a family of chromatin-remodeling enzymes that consist of one of two mutually exclusive ATPases called Brahma or Brahma-related gene 1 (Brg1) (4–6) and at least 11 additional subunits that are assembled in a seemingly limitless array of combinations that is believed to confer context- and cell type-specific functions (7, 8).

In addition to subunit composition dictating function, emerging evidence demonstrates that subunits of the complex are responsive to different signal pathways and undergo post-translational modifications that influence function (9–13). For instance, the Baf60c subunit is phosphorylated by the p38 mitogen-activated protein kinase to allow the assembly of the rest of the remodeling complex at myogenic promoters (10, 14). The Brg1 ATPase is necessary for primary myoblast proliferation and for skeletal muscle differentiation (9, 15, 16). Our group has recently shown that Brg1 is phosphorylated by protein kinase C β1 (PKCβ1), which represses Brg1 function and myogenesis (9). We also found that calcineurin is the phosphatase that opposes PKCβ1 phosphorylation of Brg1 to allow chromatin remodeling and differentiation (9). Moreover, replacement of endogenous Brg1 with the phosphomimetic Brg1 mutant inhibited myogenesis, whereas replacement with a non-phosphorylatable mutant allowed myogenesis independently of calcineurin signaling (9). In other systems, insulin signaling induces phosphorylation of Baf60c in liver as a requisite step in lipogenic gene activation (17), and the DPF/Baf45c subunit becomes phosphorylated in cardiac muscle in response to hypertrophic signaling (11). Changes in Brg1 phosphorylation have been noted in ovarian tumors (18), whereas ataxia telangiectasia-mutated kinase phosphorylates Brg1 in response to DNA-damage signaling (19). It therefore appears that the mammalian SWI/SNF enzyme complex may be a target of multiple signal transduction pathways in a context-dependent manner.

Mass spectrometry analysis of Brg1 in the presence and absence of a calcineurin inhibitor not only identified phosphorylated peptides dependent upon calcineurin but also additional phosphopeptides that did not change due to calcineurin inhibition (9), suggesting that other phosphatases and kinases also regulate Brg1 during myogenesis and almost certainly in other cell types. Further analysis of Brg1 sequence with NetPhosK predictor program (20) showed casein kinase 2 (CK2)^2^ as a putative kinase for Brg1 (9). CK2 is a ubiquitously expressed Ser/Thr kinase with over 300 identified substrates (21, 22). It is expressed as a tetrameric complex consisting of two catalytic subunits, CK2α or CK2α′, and a dimer of CK2β regulatory subunits (22). CK2 is implicated in cell survival, cell cycle, apoptosis, and transcriptional regulation. Mice lacking the α subunit of CK2 develop cardiac and neural tube defects that lead to death during embryogenesis; deletion of the α′ catalytic subunit specifically impairs spermatogenesis (23, 24). Knock-out of the β subunit causes post-implantation lethality, and conditional knock-out indicates a requirement for CK2β in cell viability (25). Similarly, in cultured cells, inhibition of CK2 leads to blockage of cell cycle progression and death (26–28). Here, we explore a possible role for CK2 in regulation of Brg1 during skeletal muscle proliferation and differentiation.

The ability of skeletal muscle to grow and to regenerate in response to injury relies on the resident stem cell population, termed satellite cells. Satellite cells are located under the basal lamina surrounding the myofibers (29). Under homeostatic conditions, satellite cells have a slow rate of turnover and are maintained in a non-proliferative state (30–32). To preserve the functional satellite cell pool, fine-tuned regulation of transcription must exist. Relevant for the myogenic lineage is Pax7, a transcriptional regulator required for proliferation of the muscle satellite cell pool (31–35). Deletion of *Pax7* in mice leads to death within 2–3 weeks after birth due to failure of developing neural crest derivatives (36). *Pax7* knock-out mice have a reduced satellite cell pool, a progressive loss of the existing satellite cell pool, and impaired capabilities to regenerate muscle (37–39). Our group recently showed that *Pax7* is a target gene of Brg1 in proliferating myoblasts (15). Brg1 binds to the *Pax7* promoter, locally remodels chromatin, and enhances *Pax7* expression. Reintroduction of *Pax7* into primary myoblasts lacking Brg1 rescues the cells from apoptosis and almost completely restores proliferation (15). These data support a mechanism whereby Brg1 regulates *Pax7* to promote cell survival and proliferation. In this work, we present new evidence that CK2-dependent phosphorylation affects Brg1 chromatin remodeling activity and, consequently, the transcription of *Pax7*. We describe a novel mechanism by which CK2 regulates Brg1-dependent myoblast proliferation by influencing the subunit composition of the SWI/SNF complex, its sub-nuclear localization, and its chromatin remodeling properties at the *Pax7* promoter. Our results suggest that regulation of the phosphorylation state of Brg1 contributes to the regulation of subunit composition of the mammalian SWI/SNF complex. We hypothesize that determination of the myoblast fate requires a dynamic equilibrium between phosphorylation and dephosphorylation of the chromatin-remodeling enzyme. Moreover, this work further supports the concept that the mammalian SWI/SNF complex acts as an integrator for multiple signaling pathways to regulate myogenesis.

## Results

### Brg1 is phosphorylated by CK2 in vitro

**Figure 1.**
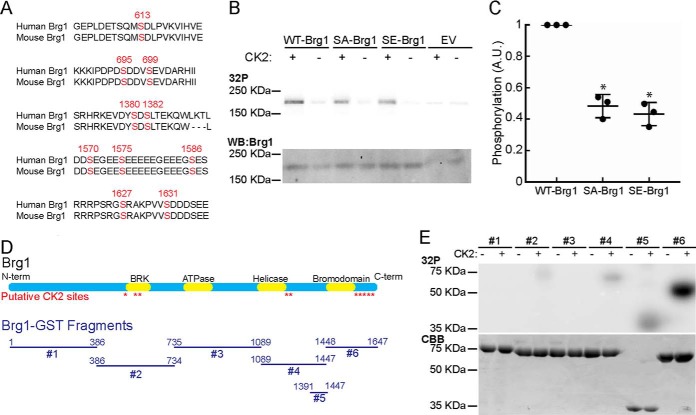
**Brg1 is phosphorylated *in vitro* by CK2.** *A,* schematic representation of the location of the putative sites for CK2 phosphorylation identified by NetphosK (9, 20). *B,* representative autoradiograms of *in vitro* CK2-treated WT-, SA-, and SE-Brg1 (*top*). A representative Western blot detecting Brg1 is shown for comparison (*bottom*). *EV,* empty vector. *C,* Brg1 labeling was quantified by normalizing autoradiography signals to the Western blotting for Brg1. Data represent the average of three independent experiments ± S.D.; *, *p* < 0.01. Phosphorylation in the WT-Brg1-expressing cells was set at 1. *A.U.*, arbitrary units. *D,* diagram of the GST-Brg1 fragments (*#1–6*) used to characterize the phosphorylation by CK2 relative to the predicted CK2 sites and the known domains of Brg1. *E,* representative autoradiogram of *in vitro* CK2-labeled GST-Brg1 fragments (*top*). Representative Coomassie Brilliant Blue (*CBB*) staining is shown for comparison (*bottom*).

Our group has shown that the regulation of Brg1 phosphorylation by PKCβ1 and the phosphatase, calcineurin, controls the ability of Brg1 to promote differentiation of cultured primary and immortalized myoblasts (9). In the course of that work, analysis of putative phosphorylation sites in Brg1 suggested that CK2 might phosphorylate Brg1 (9). Sequence analysis using the NetphosK program for predicting kinase sites (9, 20) showed 10 potential phosphorylation sites for CK2 in the murine Brg1-coding region that are conserved in the human sequence (Fig. 1*A*). To investigate whether Brg1 could be phosphorylated by CK2 *in vitro*, we mutated these 10 serine residues to non-phosphorylatable alanine (SA-Brg1) or phosphomimetic glutamate (SE-Brg1). Wild type (WT-), SA-, and SE-Brg1 were expressed via an *in vitro* transcription/translation protocol and purified by immunoprecipitation (Fig. 1*B*) with a polyclonal rabbit antisera against Brg1 (40). Purified Brg1 proteins were tested for phosphorylation in the presence or absence of purified CK2. Autoradiograms showed that WT-Brg1 was phosphorylated by CK2 (Fig. 1*B*). CK2-dependent phosphorylation of the SA- and SE-Brg1 mutants was quantified and normalized to the phosphorylation intensity of WT-Brg1 (Fig. 1, *B* and *C*). A 50% decrease in the phosphorylation of mutant SA- or SE-Brg1 was detected (Fig. 1*C*). The remaining phosphorylation indicates the presence of other unidentified CK2 target residues. To further characterize the Brg1 phosphorylation that was observed, we expressed and purified glutathione *S*-transferase (GST)-Brg1 fusions spanning the N to C termini of Brg1 (Fig. 1*D*) (9). The fragment that contains the BRK domain (fragment 2) and the C-terminal fragments (fragments 4–6) that flank or contain the bromodomain were phosphorylated by CK2 *in vitro* (Fig. 1*E*). Phosphorylation of fragment 5 indicates the presence of unidentified target amino acids. Fragment 6 was heavily phosphorylated, suggesting CK2 phosphorylation occurs predominantly at the C-terminal end of Brg1. A representative Coomassie Brilliant Blue-stained gel shows the purity of the purified GST-Brg1 fragments and is included as a loading control (Fig. 1*E*).

**Figure 2.**
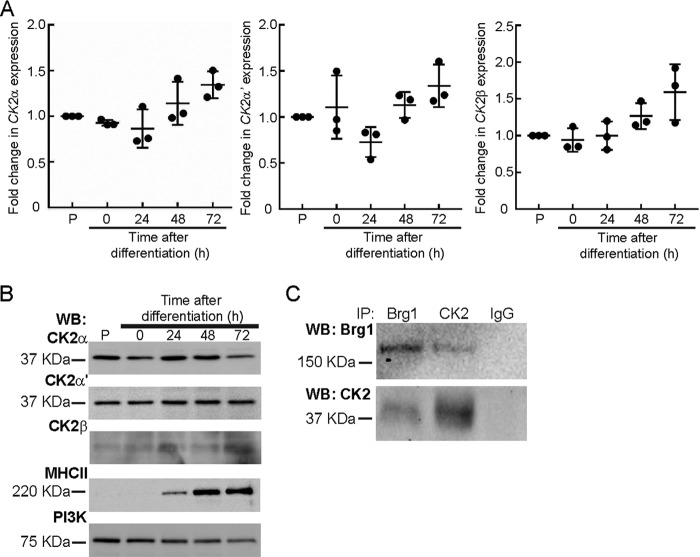
**Brg1 and CK2 interact in primary myoblasts.** Gene (*A*) and protein (*B*) expression of CK2α, CK2α′, and CK2β do not change over the course of differentiation of primary myoblasts. Expression in proliferating (*P*) primary myoblasts was set at 1. Data represent the average of three independent experiments, each assayed in triplicate ± S.D. Observed differences were not statistically significant. MHC II levels are shown as a differentiation control, and PI3K levels are shown as a loading control for the Western blots (*WB*). *C,* Brg1 and CK2 interact in proliferating primary myoblasts. A representative Western blot of the reciprocal immunoprecipitations (*IP*)of Brg1 and CK2α is shown. Pulldown with IgG was used as a negative control.

Given our prior interest in the consequences of Brg1 phosphorylation during skeletal muscle differentiation, we used cultured proliferating primary myoblasts derived from mouse satellite cells to extend our investigation. We first characterized the expression of CK2 subunits in proliferating and differentiating myoblasts. mRNA (Fig. 2*A*) and protein levels (Fig. 2*B*) of the CK2α, α′, and β subunits were largely unchanged. Considering that Brg1 was phosphorylated by CK2 *in vitro*, we asked whether an interaction between the two proteins might occur in cells. Reciprocal co-immunoprecipitation experiments showed an interaction between CK2 and Brg1 in proliferating primary myoblasts (Fig. 2*C*). These results indicate that Brg1 and CK2 can interact in proliferating myoblasts and suggest that CK2-mediated phosphorylation of Brg1 may occur via interaction of the two proteins.

### Phosphomimetic mutation of putative CK2 sites in Brg1 inhibits primary myoblast proliferation and leads to cell death, whereas CK2 inhibition increases myoblast proliferation

To assess the functionality of CK2-mediated phosphorylation of Brg1, we utilized a system we previously described to replace endogenous Brg1 with a wild type or mutated version (9). Briefly, satellite cells were isolated from skeletal muscle tissue of mice homozygous for a conditional allele encoding the Brg1 protein (Brg1 c/c) (41) and propagated in culture. The proliferating myoblasts were infected with adenovirus encoding the Cre recombinase (Ad-Cre) or an empty vector as a negative control; retroviruses encoding the described yellow fluorescent protein (YFP)-labeled WT-SA- or SE-Brg1 mutations were also introduced. The YFP-Brg1 was positioned upstream of an mCherry-coding sequence flanked by Cre recombinase sites. Therefore, in the absence of Ad-Cre, the endogenous Brg1 was retained, and mCherry, but not YFP-Brg1, was expressed from the retrovirus. In the presence of Ad-Cre, the endogenous Brg1 was deleted, and the exogenous YFP-Brg1 was expressed.

**Figure 3.**
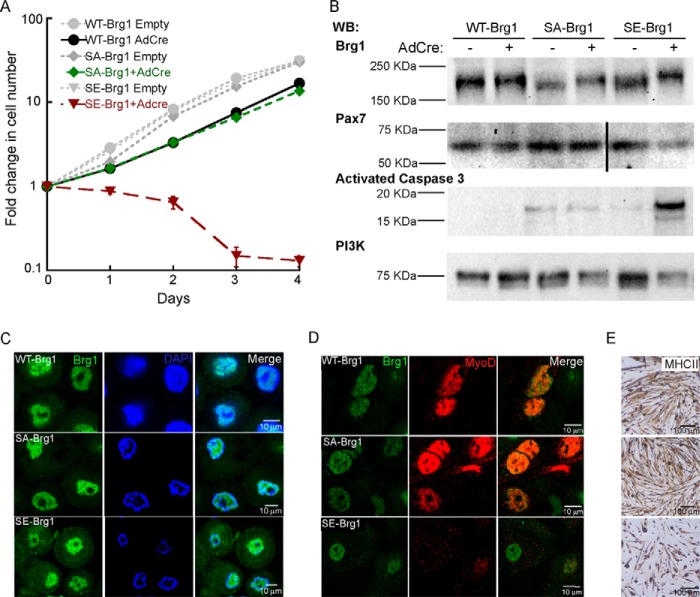
**Phosphomimetic mutations in Brg1 inhibit proliferation and reduce viability of primary myoblasts.** *A,* proliferation assay of Brg1-deficient (Brg1 c/c) cells transduced with WT-, SA-, or SE-Brg1. Data represent the average of three independent experiments ± S.D. *B,* representative Western blots (*WB*) showing the expression of Brg1, Pax7, activated caspase 3, and PI3K as a loading control. *C,* representative confocal microscopy images showing nuclear localization of WT-, SA-, and SE-Brg1 expressed in proliferating primary myoblasts. *D,* representative confocal microscopy images of primary myoblasts transduced with the WT-, SA-, and SE-Brg1 mutants that were differentiated for 48 h and immunostained for Brg1 and MyoD. *E,* representative light microscopy images of the phenotypes observed when myoblasts expressing the indicated Brg1 protein were differentiated for 48 h; cells were immunostained using an anti-myosin heavy chain II (*MHCII*) antibody.

We first evaluated cell proliferation via cell counting. All cells infected with empty adenovirus and retrovirus encoding YFP-tagged Brg1 proteins proliferated with similar kinetics (endogenous Brg1 expressed, exogenous Brg1 not expressed; *gray lines*, Fig. 3*A*). We previously demonstrated that deletion of Brg1 in primary myoblasts resulted in inhibition of proliferation and cell death and that exogenous expression of wild-type Brg1 rescued the viability and proliferation phenotypes (15). Consistent with these prior results, Ad-Cre-treated cells ectopically expressing WT-Brg1 showed growth kinetics similar to the untreated controls (Fig. 3*A*). Substitution of the 10 putative CK2 phosphorylation sites in Brg1 with alanine (SA-Brg1) rescued cell viability and proliferation similar to that observed in WT-Brg1-expressing cells. In contrast, the phosphomimetic mutant, SE-Brg1, showed an inhibition of proliferation and a subsequent loss of cell number (Fig. 3*A*). The phenotype of the SE-Brg1-expressing cells mirrored that of primary myoblasts deleted for Brg1 (15). The results suggest that phosphorylation of Brg1 by CK2 negatively impacts myoblast proliferation and viability. Expression of the exogenous WT-, SA-, and SE-Brg1 upon Ad-Cre infection was verified by Western blotting, which is evident by the increase in the molecular weight of the YFP-tagged Brg1 constructs compared with the endogenous Brg1 (Fig. 3*B*). The SE-Brg1-expressing cells showed a decrease in expression of the transcription factor Pax7, which is required for myoblast proliferation (15, 31–39, 42), and an increase in activated caspase 3 expression, which indicates apoptosis (Fig. 3*B*) (15, 43). These results at least partially explain the proliferation defect and loss of viability of the SE-Brg1 mutant. Nuclear localization of Brg1 wild-type and mutant forms was verified by confocal microscopy, although the SE-Brg1 mutant also showed some cytoplasmic staining (Fig. 3*C*).

To assess a role for CK2-dependent phosphorylation of Brg1 in myogenesis, we induced the primary myoblasts to differentiate. Cells expressing the WT-Brg1 and the SA-Brg1 mutant expressed MyoD (Fig. 3*D*) and differentiated, as shown by myotube formation and expression of the differentiation marker myosin heavy chain II (*MHCII*, Fig. 3*E*). Cells expressing the SE-Brg1 mutant showed limited expression of the myogenic regulatory factor MyoD and the differentiation marker MHCII and were unable to differentiate. Failure of the SE-Brg1 mutant cells to differentiate likely can be attributed to the fact that cells were undergoing apoptosis.

**Figure 4.**
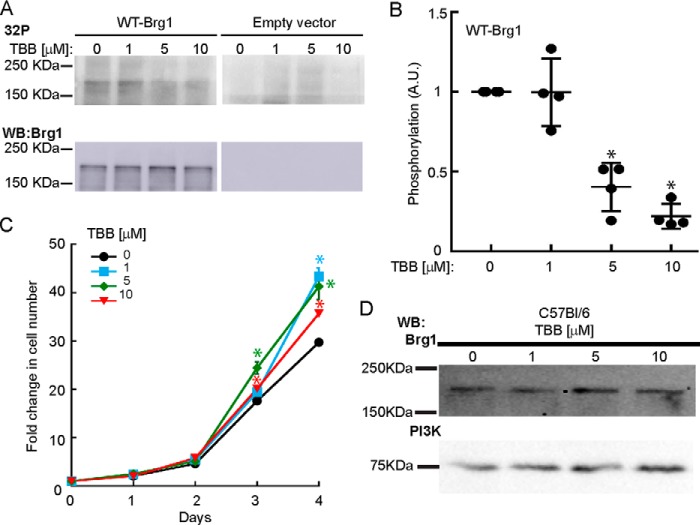
**Inhibition of CK2 affects CK2-mediated *in vitro* phosphorylation of Brg1 and primary myoblast proliferation.** *A,* dose-dependent inhibition of CK2-mediated *in vitro* phosphorylation of Brg1 by TBB. Representative autoradiograms (*top*) and Western blots (*WB*) confirming the expression of Brg1 (*bottom*) are shown. *B,* quantification of phosphorylation changes presented as the mean ± S.D. from four independent experiments *, *p* < 0.0002. *C,* proliferation assay of C57Bl/6 myoblasts treated with increasing concentrations of the CK2 inhibitor TBB. Data represent the average of three independent experiments ± S.D. *, *p* < 0.01. *D,* representative Western blots of Brg1 expression in primary myoblasts. PI3K was examined as a loading control.

Because mimicking the effect of CK2 kinase activity on Brg1 reduced myoblast proliferation, inhibition of CK2 might be expected to increase myoblast proliferation. However, CK2 has many target proteins; attempts to knock down CK2 by RNA inhibition have largely resulted in growth inhibition and cell death (21, 22). Therefore, we investigated the effect of 4,5,6,7-tetrabromobenzotriazole (TBB), a highly specific inhibitor of the ATP-binding site in CK2 (44), on CK2-mediated phosphorylation of Brg1 and on myoblast proliferation and viability. *In vitro* transcribed/translated Brg1 was mixed with CK2 enzyme *in vitro*, and increasing amounts of TBB were added to the reaction. Inhibition of Brg1 phosphorylation was observed at 5 and 10 μm TBB (Fig. 4, *A* and *B*). Proliferating myoblasts derived from C57Bl/6 satellite cells were incubated with increasing concentrations of TBB for 4 days, and the proliferation rate was evaluated. Fig. 4*C* shows an increase in the proliferation of myoblasts when treated with sub-lethal concentrations of TBB (1–10 μm). Consistent with other cell types, TBB was toxic to the myoblasts at concentrations above 50 μm (44, 45). Brg1 expression was not affected by TBB treatment (Fig. 4*D*). Overall, these results suggest that a functional consequence of phosphorylation of Brg1 by CK2 is to regulate myoblast proliferation.

### Phosphomimetic mutation of putative CK2 sites in Brg1 inhibits proliferation and viability by preventing chromatin remodeling at and expression from the Pax7 promoter

**Figure 5.**
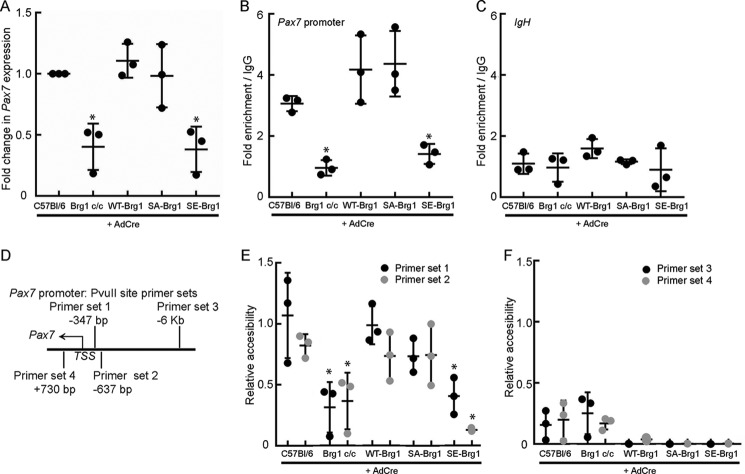
**Phosphomimetic Brg1 mutant does not bind to or remodel chromatin at the *Pax7* promotor.** *A,* mRNA expression levels of *Pax7* in the indicated primary myoblasts. Values for the C57Bl/6 primary myoblasts were set at 1. *B* and *C,* quantification of ChIP assays measuring binding of Brg1 to the *Pax7* promoter (*B*) or the *IgH* enhancer (*C*) in each of the indicated primary myoblasts. *D,* schematic representation of the location of the PvuII sites and the primer sets used for REAA. *E* and *F,* quantification of REAAs performed on the indicated primary myoblasts using primer sets 1 and 2 (*E*) or primer sets 3 and 4 (*F*). The accessibility at the PvuII site assessed by primer set 1 in the WT-Brg1-expressing primary myoblasts was set at 1. All data represent the average of three independent experiments, each assayed in triplicate ± S.D.; *, *p* < 0.01.

We previously showed that Brg1 regulates chromatin accessibility at and expression from the promoter of the transcriptional regulator *Pax7*, which is necessary for satellite cell proliferation and survival under non-differentiating conditions (15, 31–39, 42). To better understand the effect of CK2 phosphorylation of Brg1, we examined *Pax7* expression in Brg1-deficient cells expressing WT-Brg1, SA-Brg1, or SE-Brg1. We observed a decrease in Pax7 protein expression in proliferating SE-Brg1- but not in the SA-Brg1-expressing myoblasts compared with the WT control (Fig. 3*B*). Consistent with these results, *Pax7* mRNA levels were decreased in SE-Brg1-expressing cells, indicating a deficiency at the level of transcription (Fig. 5*A*). We then analyzed whether or not this phenotype was due to mechanistic deficiencies in the chromatin-binding or chromatin-remodeling properties of the phosphomimetic Brg1. Chromatin immunoprecipitation (ChIP) assays revealed binding of Brg1 to the *Pax7* promoter region relative to the IgG control in proliferating myoblasts expressing WT-Brg1 or SA-Brg1 (Fig. 5, *B* and *C*). ChIP results also indicated that myoblasts expressing SE-Brg1 showed little or no binding of Brg1 to the *Pax7* promoter, matching levels observed in cells deleted for Brg1 and levels observed at the *IgH* enhancer, which was used as a negative control sequence (Fig. 5, *B* and *C*). These results suggest that the interaction between Brg1 and the *Pax7* promoter can be blocked by CK2-mediated hyperphosphorylation of Brg1. The lack of SE-Brg1 binding to the *Pax7* promoter suggests a deficiency in chromatin remodeling at the promoter, as we have previously demonstrated that Brg1 contributes significantly to *Pax7* promoter accessibility in proliferating myoblasts (15). Restriction enzyme accessibility assays (REAA) utilizing two PvuII cleavage sites within the *Pax7* promoter region (Fig. 5*D*) showed a significant increase in the accessibility at two nuclease sites in the promoter region of *Pax7* that are 347 and 637 bp upstream of the transcription start site in the WT- and SA-Brg1-expressing cells (*primer sets 1* and *2*, Fig. 5*E*). As expected, deletion of Brg1 in the conditional myoblasts led to a significant decrease in nuclease accessibility at the *Pax7* promoter, and accessibility in the presence of SE-Brg1 was similarly inhibited (Fig. 5*E*). No PvuII cleavage was detected at the restriction site located 6 kb upstream from the *Pax7* promoter or at a site within the coding sequence (*primer sets 3* and *4*, Fig. 5*F*) in each of the cells, reflecting inaccessibility to this nuclease in these regions. Together, these results suggest that the hyperphosphorylated state of Brg1 abolishes its binding to the *Pax7* promoter, reducing its capability for chromatin remodeling, and therefore reducing *Pax7* gene and protein expression and leading to a loss of myoblast viability.

### Intra-nuclear mobility and localization of Brg1 is altered in the SA- and SE-Brg1 mutants

**Figure 6.**
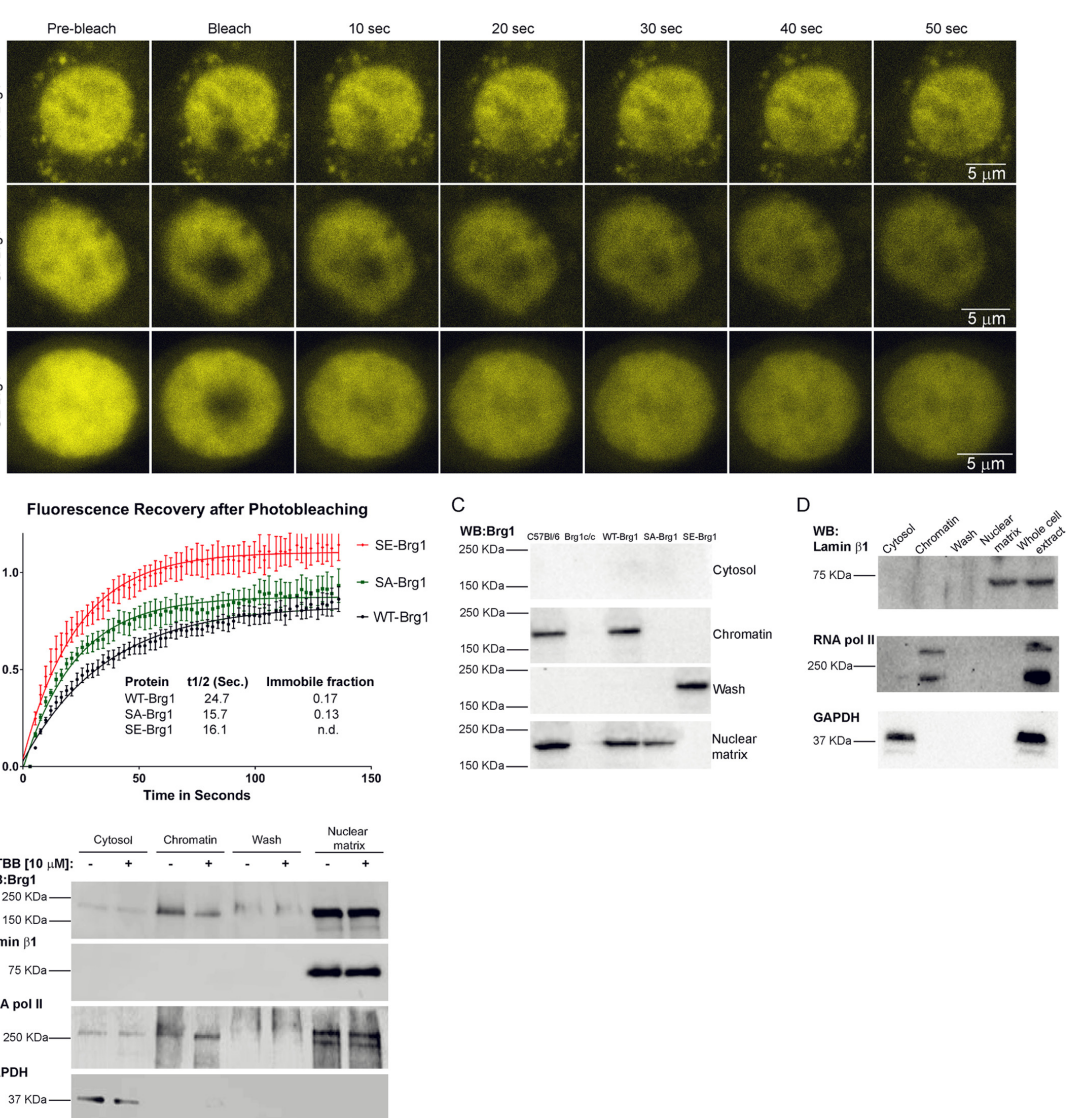
**Phosphomimetic and non-phosphorylatable mutations in Brg1 or inhibition of CK2 affects intranuclear mobility and association with chromatin and the nuclear matrix.** *A,* time course of representative confocal microscopy images from FRAP experiments performed on primary myoblasts expressing WT-, SA-, or SE-Brg1. *B,* quantification of the fluorescence recovery of WT-, SA-, and SE-Brg1 mutants. Data represent the average values from five independently examined cells ± S.E. *n.d.,* not detected. *C,* representative Western blottings (*WB*) of Brg1 in the indicated fractions derived from proliferating primary myoblasts. *D,* representative Western blottings from C57Bl/6 myoblasts for marker proteins to demonstrate the purity of the fractions used in *C. E,* representative Western blots of Brg1 and marker proteins present in the indicated fractions from primary myoblasts treated with TBB.

Because the hyper-phosphorylated version of Brg1 was not able to remodel chromatin at the *Pax7* promoter, and thus caused deficient proliferation and increased apoptosis, we tested whether this protein has altered localization within the nucleus and reduced binding to chromatin and/or the nuclear matrix. First, taking advantage of the YFP labeling of the exogenous WT-, SA-, and SE-Brg1 alleles, we performed fluorescence recovery after photobleaching (FRAP). After photobleaching a region of interest (3 μm diameter) in the nucleus, we observed half-time recoveries on the order of 15–25 s (Fig. 6, *A* and *B*). FRAP measures binding and diffusion (46), and because diffusion-limited recovery is very fast, on the order of tenths of seconds (47), we were measuring binding in these experiments. The initial recovery of fluorescence was faster for the myoblasts expressing the SE-Brg1 construct compared with the WT-Brg1, whereas the immobile fraction was smaller (Fig. 6, *A* and *B*). The initial recovery rate measures the binding that is in more rapid equilibrium, whereas the immobile fraction measures tight binding that did not recover during the 140-s time course of the experiment. Thus, there are at least two pools of BRG1, differing in binding constants and both affected by the phosphomimetic mutation. These results suggest that the phosphomimetic Brg1 mutant has increased mobility compared with the wild-type and non-phosphorylatable Brg1. Surprisingly, the SA-Brg1 molecule, which cannot be phosphorylated, showed different binding properties compared with the SE- and WT-Brg1 proteins. The initial recovery rate half-time was similar to that of the SE-Brg1 protein, but the immobile fraction was similar to that of the WT-Brg1 protein, suggesting that despite the ability of the SA mutant to support chromatin remodeling and expression at the *Pax7* promoter and myoblast proliferation, the alteration in phosphorylatable serines changed its physical properties and its binding properties in live cells.

Nuclear fractionation experiments have shown that mammalian SWI/SNF proteins are associated with the nuclear matrix, also called the nuclear scaffold or the nucleoskeleton (48, 49). The nuclear matrix is the non-chromatin, fibrogranular ribonucleoprotein network that exists in the nucleus (50) and serves to organize chromatin loops by providing an anchoring point at the base of the loop (51). Because both the phosphomimetic and the non-phosphorylatable Brg1 mutants exhibited altered nuclear mobility, we hypothesized that the intra-nuclear distribution of the mutant proteins might be affected. To test this hypothesis, we performed a sequential nuclear extraction procedure that previously revealed the association of Brg1 with the nuclear matrix (48, 52). This fractionation begins with the extraction of cytoplasmic proteins with Triton X-100. Chromatin proteins then were released by DNase I digestion. Samples were washed with high salt buffer, leaving a fraction containing the nuclear matrix proteins and nuclear matrix-associated proteins. Fig. 6*C* shows a representative Western blot of the four fractions obtained for all the primary myoblasts used. Parental primary myoblasts showed endogenous Brg1 localized to the chromatin and to the nuclear matrix in roughly equal proportions, a result also observed in Brg1-deleted myoblasts ectopically expressing WT-Brg1 (Fig. 6*C*) and in other cell types (48, 52). The SE-Brg1 mutant was found in the high-salt wash fraction (Fig. 6*C*), associated with neither chromatin nor with the nuclear matrix, likely explaining its inability to bind to the *Pax7* promoter. Interestingly, the SA-Brg1 mutation protein was exclusively found in the nuclear matrix fraction (Fig. 6*C*). This result is consistent with the FRAP data showing that the immobile fraction of the SA-Brg1 mutant was similar to that of WT-Brg1. The lack of a chromatin-associated fraction may relate to the difference in initial recovery half-time relative to the WT-Brg1. Additional thoughts on how the SA-Brg1 mutant functions like the WT-Brg1 in promoting *Pax7* expression, despite not being associated with the soluble chromatin fraction, are presented under the “Discussion.” Representative control Western blottings show the purity of the fractions (Fig. 6*D*). Lamin β1 is only localized to the nuclear matrix, RNA pol II is associated with the chromatin and the nuclear matrix, and GAPDH is located only in the cytosolic fraction.

To complement these results, we examined sub-nuclear localization of Brg1 from cells treated with the CK2 inhibitor TBB. TBB treatment reduced the level of Brg1 in the chromatin, but not the nuclear matrix fraction (Fig. 6*E*), consistent with the depletion of Brg1 in the chromatin fraction when the non-phosphorylatable SA-Brg1 mutant was expressed (Fig. 6*C*). TBB treatment also increased the gel mobility of chromatin-associated Brg1, as would be expected if phosphates were removed from a phosphoprotein (Fig. 6*E*). Interestingly, the gel mobility of the nuclear matrix-associated Brg1 is equivalent to that of the TBB-treated, chromatin-associated Brg1, suggesting that the nuclear matrix-associated Brg1 is also relatively less phosphorylated than the chromatin-associated Brg1. The reduced phosphorylation state likely links with the ability of Brg1 to promote *Pax7* transcription (see “Discussion”). We noted similar changes in gel mobility for RNA pol II; the chromatin-associated RNA pol II present in TBB-treated cells showed increased gel mobility, similar to the increased mobility observed for RNA pol II in the nuclear matrix, with or without TBB treatment. Prior reports indicate that CK2 interacts with and phosphorylates RNA pol II (53–56). Collectively, these data suggest that CK2-dependent phosphorylation of Brg1 determines the sub-nuclear localization of this chromatin-remodeling enzyme and specifically its association with chromatin and the nuclear matrix.

### Mutation of Brg1 alters association with other subunits of the mammalian SWI/SNF complex

We asked then whether or not the association of Brg1 with other members of the enzyme complex might be dependent in the phosphorylation state of Brg1. The Baf170 and Baf155 subunits show greater than 60% identity (57) and are preferentially incorporated into SWI/SNF complexes in certain cell types (7, 8). Specifically, mouse embryonic stem cells (ESCs) predominantly incorporate Baf155 into enzyme complexes (58). Upon neuronal differentiation, Baf170 expression is induced, whereas Baf155 expression decreases, and there is an increase in the amount of SWI/SNF complexes that incorporate Baf170 (58). We therefore investigated whether Baf170 and Baf155 association with Brg1 was affected by the SA or SE mutations.

**Figure 7.**
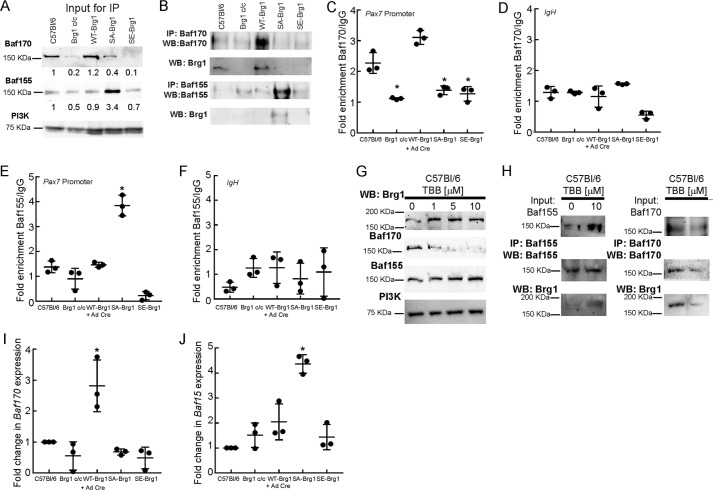
**Inhibition of CK2 or expression of phosphomimetic or non-phosphorylatable Brg1 mutations determines interaction with Baf170 or Baf155.** *A,* representative Western blots (*WB*) showing 12% of the input for the subsequent immunoprecipitation (*IP*) experiments, which documents expression of Baf170 and Baf155 in the indicated proliferating primary myoblasts. Bands were quantified using ImageJ. Brg1 expression was normalized to PI3K expression, and the value of expression in the C57Bl/6 myoblasts was set at 1. Relative expression levels are indicated below each band. *B,* representative profiles of Baf170 and Baf155 immunoprecipitations of Brg1 from the indicated primary myoblasts. *C* and *D,* ChIP experiments showing Baf170 recruitment to the *Pax7* promoter (*C*) or to the *IgH* enhancer (*D*) when endogenous or WT-Brg1 is expressed. *E* and *F,* ChIP experiments showing Baf155 recruitment to the *Pax7* promoter (*E*) or to the *IgH* enhancer (*F*) when the non-phosphorylatable Brg1 mutant is expressed. Data in *C–F* represent the average of three independent experiments, each assayed in triplicate ± S.D.; *, *p* < 0.01. *G,* representative Western blottings showing the expression of Baf170, Baf15, and Brg1 in C57Bl/6 primary myoblasts treated with increasing concentrations of TBB. PI3K levels were measured as a control. *H,* representative immunoprecipitations of Baf155/Brg1 and Baf170/Brg1 from primary myoblasts treated with 10 μm TBB. *I* and *J,* relative levels of Baf170 and Baf155 mRNA present in the indicated myoblasts. Levels in C57Bl6 primary myoblasts were normalized to 1. Data represent the average of three independent experiments, each assayed in triplicate ± S.D.; *, *p* < 0.01.

First, we analyzed whether the expression of SA- or SE-Brg1 had an effect on Baf155/Baf170 protein levels. Parental and WT-Brg1-expressing myoblasts expressed Baf170 protein at roughly equivalent levels. Baf170 levels were significantly reduced in Brg1-deleted cells as well as in the SE-Brg1-expressing cells, although the SA-Brg1-expressing cells showed an approximate 2-fold reduction (Fig. 7*A*). In contrast, Baf155 levels were only modestly reduced in the SE-Brg1-expressing cells (30% reduction) and in the Brg1-deleted cells (50% reduction), although the SA-Brg1-expressing cells showed a greater than 3-fold increase in Baf155 levels (Fig. 7*A*). Reciprocal immunoprecipitations showed that the Brg1 isolated from parental and WT-Brg1-expressing myoblasts preferentially interacted with Baf170, whereas the non-phosphorylatable SA-Brg1 mutant preferentially interacted with Baf155 (Fig. 7*B*), which is consistent with the levels of expression of these proteins. To further analyze subunit association, we analyzed the binding of Baf170 and Baf155 to the *Pax7* promoter by ChIP. Consistent with the immunoprecipitation results, Baf170 can be localized at the *Pax7* promoter in parental and WT-Brg1-expressing cells (Fig. 7, *C* and *D*), whereas Baf155 only associates with the *Pax7* promoter in SA-Brg1-expressing cells (Fig. 7, *E* and *F*). The results indicate that Brg1 normally associates with Baf170 in proliferating myoblasts; the non-phosphorylatable Brg1 switches to associate with Baf155, and the phosphomimetic SE-Brg1 interacts poorly with either Baf170 or Baf155.

Treatment with the CK2 inhibitor TBB provided corroborating results. TBB treatment inhibited Baf170 expression and modestly increased Baf155 expression (Fig. 7*G*). Immunoprecipitation of Baf155 from cells treated with TBB showed an increased level of Brg1 associated with Baf155 compared with the association in untreated cells, whereas immunoprecipitation of Baf170 from TBB-treated cells showed decreased association of Brg1 with Baf170 compared with the untreated cells (Fig. 7*H*). Thus CK2 inhibition decreased interaction between Brg1 and Baf170 while favoring the association of Brg1 with Baf155.

Modification of amino acids in Brg1 that are targets for phosphorylation could affect interaction with other SWI/SNF subunits by directly altering the affinity of or the potential for protein–protein interactions. Alternatively, the effect could be more indirect. To gain insight into this question, we examined Baf170 and Baf155 mRNA levels in control and Brg1-deleted cells expressing WT, SA-, or SE-Brg1. Baf170 mRNA levels (Fig. 7*I*) largely matched Baf170 protein levels (Fig. 7*A*). We note that WT-Brg1-expressing cells showed higher levels of Baf170 mRNA than the parental cells expressing endogenous Brg1, but this increase did not translate to significantly increased Baf170 protein levels (Fig. 7, *I* and *A*). Similarly, the Baf155 mRNA levels in each of the different cells largely matched the Baf155 protein levels. Notably, the increase in Baf155 protein levels was mirrored by an increase in Baf155 mRNA (Fig. 7, *J* and *A*). These data suggest that the preferential association between the SA-Brg1 mutant and Baf155 in cells treated with the CK2 inhibitor may be a result of changes in the expression of the genes that encode Baf155 and Baf170, which then alters the relative availability of these subunits for SWI/SNF complex assembly. The data do not exclude an effect of phosphorylation on protein–protein interactions; instead, the findings suggest that association of different SWI/SNF subunits may be regulated by multiple mechanisms.

## Discussion

### Brg1 is an in vitro substrate for CK2

*In silico* analysis of the Brg1 protein sequence showed 10 residues that are potential targets for CK2-dependent phosphorylation (Fig. 1*A*). CK2 phosphorylates Ser and Thr residues that are in proximity to acidic residues (*i.e.* Ser-Xaa-Xaa-acidic, where the acidic residue may be Glu, Asp, phospho-Ser, or phospho-Tyr). There are constraints associated with using this consensus sequence for the identification of CK2 targets, as the presence of this sequence does not guarantee phosphorylation, and there may be other determinants within the sequence that modulate phosphorylation efficiency (22, 59). It is also possible that certain sequences do not fit in this consensus sequence but are effectively phosphorylated by CK2, as in the case of the tumor suppressor p53 (60). *In vitro* phosphorylation experiments using purified Brg1 confirmed that this ATPase is a substrate of CK2. Purified Brg1 containing non-phosphorylatable and phosphomimetic mutations at the identified potential CK2 sites showed decreased phosphorylation by CK2. This partial decrease in phosphorylation is likely due to the presence of other unidentified CK2 target residues elsewhere in the Brg1-coding sequence. Our experiments with GST-Brg1 constructs (Fig. 1*E*) showed that the fragment flanking the bromodomain (construct 6) appeared to be heavily phosphorylated compared with other fragments. Whether this indicates additional CK2 sites in this region or simply reflects a strong preference for the predicted sites that are present remains unclear. The results also reflect the activity of purified CK2 on recombinant Brg1. Additional differences in phosphorylation may exist due to the incorporation of Brg1 into the SWI/SNF enzyme complex.

The argument for CK2 as a relevant kinase for the mammalian SWI/SNF enzyme is supported by recent evidence showing that CK2 phosphorylates DPF3a, an isoform of a different subunit of the enzyme complex that is also called Baf45c (11). Upon signaling that induces cardiac muscle hypertrophy, CK2 phosphorylates DPF3a at a specific serine residue. The consequence is that phosphorylated DPF3a and other SWI/SNF enzyme subunits bind to chromatin at promoters encoding fetal cardiac genes that are silenced by the Hey family of transcriptional repressors, resulting in displacement of Hey factors and replacement with transcriptional activators. Phosphorylation of DPF3a was required for the interaction with the Hey repressors (11), suggesting a role for CK2-dependent phosphorylation of mammalian SWI/SNF subunits. CK2 has also been implicated as a regulator of other chromatin-remodeling enzymes. The *Drosophila* Mi-2 ATPase is phosphorylated by CK2, which increases both affinity for chromatin and *in vitro* enzymatic activity (61).

### CK2 is implicated as a kinase that modulates the ability of Brg1 to support myoblast proliferation via induction of Pax7 expression

Mutations of Brg1 that mimic the constitutive phosphorylation of these 10 residues altered the proliferation and survival of proliferating primary myoblasts. Cells expressing the SE-Brg1 mutant had a proliferation defect and underwent apoptosis, as indicated by the induction of caspase 3 (Fig. 3*B*), similar to what was observed in myoblasts deleted for Brg1 or myoblasts expressing a Brg1 catalytically inactive mutant (15). Thus, mimicking constitutive phosphorylation of Brg1 at the implicated sites renders Brg1 non-functional for its roles in promoting myoblast cell division and viability and identifies at least some of these residues as having a specific role in the regulation of myoblast proliferation. In contrast, re-introduction of the non-phosphorylatable SA-Brg1 mutant to Brg1-deleted myoblasts rescued the proliferation phenotype to the same extent as WT-Brg1. This result suggests that the inability of these sites to be phosphorylated has no impact on the ability of Brg1 to promote myoblast proliferation and viability. However, pharmacological inhibition of CK2 activity increased myoblast proliferation, suggesting that critical target residues for CK2 remain functional in the SA mutant and, more importantly, that there is a dynamic balance between the deposition and removal of phosphate groups on Brg1. Pharmacological inhibition of CK2 activity increased the rate of myoblast proliferation, although mimicking constitutive phosphorylation blocked proliferation and cell viability. A balance between phosphorylation and dephosphorylation is likely a necessity.

One possibility is that the relative level of Brg1 phosphorylation by CK2 is regulated based on the enzyme function to be performed. For example, phosphorylation state could determine whether the enzyme remodels chromatin or engages or releases from chromatin or from interacting regulatory factors. The findings about DPF3 phosphorylation discussed above provide support for such a role. An alternative explanation is that Brg1 phosphorylation by CK2 is a transitory event during proliferation. Brg1 is hyperphosphorylated during mitosis and disengages from the condensing chromatin; SWI/SNF enzymes purified from mitotic cells are deficient for *in vitro* ATP-dependent nucleosome remodeling activity (62, 63). In this scenario, CK2-mediated phosphorylation would be necessary for the inactivation of the mammalian SWI/SNF complex, all because of the myoblasts to undergo mitosis. As suggested by our data and as further discussed below, a third possibility is that dynamic regulation of Brg1 phosphorylation could be used to control interactions between different SWI/SNF subunits and therefore as a regulator of the assembly and diversity of SWI/SNF enzyme complexes. None of these possibilities is mutually exclusive.

Regardless of the exact nature of CK2's contribution to Brg1 function, it is manifested in control of *Pax7* gene expression. Pax7 is a master transcriptional regulator for the proliferative state of the muscle stem cell pool (15, 31–39, 42). Pax7 is required for the maintenance of the satellite cell population, as *Pax7* knock-out mice have a decreased number of muscle satellite cells and fail to regenerate muscle (37–39). Our studies in proliferating primary myoblasts have shown that *Pax7* regulation is driven by Brg1 (15). Deletion of Brg1 leads to a decrease in the expression of *Pax7* due to a failure of chromatin remodeling at the *Pax7* promoter region (15). ChIP and REAA presented here suggest that the interaction of Brg1 with the *Pax7* promoter relies on its phosphorylation state. The non-phosphorylatable SA-Brg1 mutant interacted with the *Pax7* promoter in a manner similar to the WT-Brg1, but chromatin interaction and remodeling were abolished when SE-Brg1 was expressed. The extent of the decrease in *Pax7* binding, in chromatin remodeling at the *Pax7* promoter, and in expression of *Pax7* mRNA due to expression of the SE-Brg1 mutant was equivalent to that observed in cells completely deleted for Brg1, suggesting that a cell expressing a Brg1 molecule that mimics constitutive phosphorylation by CK2 is equivalent to a nullizygous cell. Because introduction of *Pax7* into Brg1 null cells rescued viability and proliferation (15), the primary function of Brg1 in myoblasts is to regulate *Pax7*. It is likely, however, that a comprehensive analysis of gene expression in primary myoblasts expressing the WT-, SA-, and SE-Brg1 will identify other Brg1 target genes and will improve understanding of additional downstream consequences of CK2-dependent phosphorylation during myoblast proliferation.

In addition to effects on *Pax7* gene expression, CK2 impacts Pax7 protein stability. Pax7 levels decrease rapidly upon the induction of myoblast differentiation. Activation of the caspase 3 protease contributes to Pax7 degradation via targeted cleavage of Pax7 (64, 65). This process is impacted by CK2, which phosphorylates Pax7 in the vicinity of the caspase 3 cleavage site and sterically inhibits caspase 3 activity (64, 66, 67). CK2 phosphorylation of Pax7 therefore promotes myoblast proliferation as well as satellite cell renewal. Although mimicking constitutive phosphorylation of Brg1 through the expression of the SE-Brg1 mutant is deleterious to myoblast survival, we suggest that appropriate regulation of Brg1 phosphorylation likely supports myoblast differentiation, raising the possibility that CK2-mediated phosphorylation of Brg1 promotes *Pax7* expression, while its simultaneous phosphorylation of Pax7 promotes Pax7 protein stability, reinforcing the proliferative state in myoblasts.

### Molecular consequences of Brg1 mutation and CK2 inhibition, changes in sub-nuclear localization and alterations in SWI/SNF enzyme complex composition

To assess the binding of Brg1 in nuclear complexes in live cells and changes in binding due to Brg1 mutations that mimicked or prevented phosphorylation, we performed FRAP experiments of YFP fused to WT-, SA-, and SE-Brg1. Photobleaching experiments showed significantly decreased initial binding and a smaller fraction of tightly bound phosphomimetic SE-Brg1 compared with WT-Brg1. Subnuclear fractionation showed that endogenous myoblast Brg1, as well as the ectopically expressed WT-Brg1, were associated with both the nuclear matrix and chromatin, consistent with previous observations (48, 49). The SE-Brg1 mutant, however, was associated with neither. This is consistent with the absence of an immobile fraction of the SE-Brg1 molecule seen in live cells by FRAP; it is not anchored by any sub-nuclear structure. Interestingly, the SA-Brg1 mutant showed an immobile fraction that was similar to that of the WT-Brg1 but an initial recovery that was similar to that of the SE-Brg1 mutant. The SA-Brg1 protein associated with the nuclear matrix, but its interaction with chromatin was greatly decreased. Yet myoblasts expressing the SA-Brg1 supported proliferation and cell viability, and SA-Brg1 could be localized to the *Pax7* promoter by ChIP. This indicates that Brg1 association with the nuclear matrix is sufficient for function and also indicates that the *Pax7* promoter is associated with or in close proximity to the nuclear matrix. Prior studies looking at specific genes have generally linked matrix-associated DNA with active transcription (68–70), and the only genome-wide approach to date showed that matrix-associated DNA was enriched in actively expressing genes in immortalized but non-tumorigenic cells, although it was enriched in non-expressed genes in a cancer cell line (71). Thus our observations are consistent with the limited amount of information that exists on this subject. The biophysical reasons for the altered binding of the SA- and the SE-Brg1 mutants in live cells as measured by FRAP remain to be determined. Contributing factors likely include the number of different complexes in which Brg1 can bind, the fraction of Brg1 in each complex, the binding constants for those interactions, and the association of those complexes with the different forms of chromatin created by epigenetic modifications and with sub-structures of the nuclear matrix.

Because the SE-Brg1 mutant was detected in the high-salt wash fraction, associated with neither the chromatin nor the matrix, and was functionally equivalent to a complete loss of Brg1, we speculated that the interactions between the SE-Brg1 and other subunits of the SWI/SNF complex may be compromised or absent. Many mammalian SWI/SNF subunits are encoded by genes that encode highly similar proteins and/or generate different proteins due to alternative splicing. Some subunits are mutually exclusive or co-dependent on other subunits for inclusion in the complex. The diversity of subunit composition suggests a high degree of specialization in enzyme complex function (7, 8). An example is utilization of the Baf170 and Baf155 subunits. In mouse ESCs, the Baf155 subunit is preferentially expressed and incorporated into BRG1-based SWI/SNF complexes, whereas ESC differentiation into the neuronal lineage leads to down-regulation of Baf155, induction of Baf170, and preferential incorporation of Baf170 into Brg1-based SWI/SNF enzyme complexes (58). Assessment of Brg1 interactions with Baf155 and Baf170 showed that endogenous and WT-Brg1 preferentially interacted with Baf170, whereas the SE mutant interacted poorly but measurably with the Baf170 protein. The non-phosphorylatable SA mutant and the endogenous Brg1 from cells treated with CK2 inhibitor preferentially interacted with Baf155. We propose that CK2-mediated phosphorylation of Brg1 contributes to and perhaps drives the regulation of the subunit composition of the SWI/SNF complex and, consequently, its function.

### Signaling to mammalian SWI/SNF enzyme as a regulatory mechanism controlling myogenesis

Prior work has identified the Baf60c subunit as a substrate for the p38 MAPK. Phosphorylation of BAF60c by p38 at a specific threonine facilitates assembly of SWI/SNF enzyme at myogenic promoters and is required for myogenic differentiation (14). Phosphorylation of Brg1 at amino acids N- and C-terminal to the bromodomain by PKCβ1 prevents Brg1 from binding to or remodeling chromatin and is therefore refractory to myogenic gene expression and differentiation. Dephosphorylation by calcineurin counteracts PKCβ1 activity and removes these phosphate groups, activating Brg1 and the SWI/SNF enzyme function (9). Therefore, there are at least two signaling pathways converging on SWI/SNF enzymes that modulate their function and control myogenic differentiation. Here, we have added CK2 to the list of kinases that modify SWI/SNF subunits. In contrast, however, our evidence suggests that CK2-mediated phosphorylation affects the proliferation of myoblasts and does so by controlling sub-nuclear localization and the composition of subunits present in the enzyme complex. Thus, we have established that regulation of SWI/SNF enzyme by phosphorylation includes both the proliferation as well as the differentiation phase of myogenesis. Given the large number of subunits and the complexity of SWI/SNF enzyme composition, we suspect that additional kinases and phosphatases also impact SWI/SNF function in myogenesis and that regulation by phosphorylation is a general principle for controlling SWI/SNF enzyme composition and function in all cell types.

## Experimental procedures

### Bioinformatic analyses

Brg1 sequence was analyzed for putative CK2 phosphorylation sites using the open source NetPhosK program (9, 20).

### Plasmid preparation

The full-length human wild-type Brg1 (WT-Brg1) was cloned into pCR3.1 (Thermo Fisher Scientific) (9). Ten putative CK2 phosphorylation sites in Brg1 were mutated to alanine (non-phosphorylatable, SA-Brg1) or glutamate (phosphomimetic, SE-Brg1) using the QuikChange Lightning Multisite-directed mutagenesis kit (Agilent) and primers listed in Table 1. Mutations were verified by sequencing. The wild-type and mutant Brg1 sequences were fused to YFP by isolating the NheI–BamHI fragment from EYFP-c1–Brg1 (72) (kind gift of Dr. G. Hager, National Institutes of Health), which contains YFP fused to the N terminus of Brg1, and that fragment was cloned into AgeI–BamHI-digested pCR3.1-Brg1 for wild type and each mutant Brg1 sequence. The inducible pBabe LoxP-mCherry vector was previously described (9). This vector was digested with AgeI and PmeI as was the pCR3.1-YFP-Brg1, and the YFP WT-, SA-, and SE-Brg1 fragments were ligated to create retroviral vectors encoding the three BRG1 alleles. GST-Brg1 truncated fusions were reported previously (9).

**Table 1:** **Primers used in this study** F is forward; R is reverse.

Primer name	Sequence 5′–3′	Use
*Pax7* LM1 F	GAGATCTGAATTCCTGCGCCTTGC	REAA
*Pax7* LM1 R	GATCGAGGGGAGGAGGGAGTCC	REAA
*Pax7* LM2 F	CCGGGAGATCTGAATTCCTGGGTAAG	REAA
*Pax7* LM2 R	TCCCCTCTCCCTCCCGAACTGG	REAA
*Pax7* LM3 F	CCGGGAGATCTGAATTCCTGGAGCAA	REAA
*Pax7* LM3 R	CTCTTTCTTGAACCCAGATAGCCTGCC	REAA
*Pax7* LM4 F	CCGGGAGATCTGAATTCCTGAGGGTG	REAA
*Pax7* LM4 R	GGCTGGACAGGATCTTCA	REAA
q*Pax7* F	GCAGCTGGAGGAGCTAGAGAAG	Gene expression
q*Pax7* R	GTCTCCTGGCTTGATGGAGTCG	Gene expression
q*EF1*α F	AGCTTCTCTGACTACCCTCCACTT	Gene expression
q*EF1*α R	GACCGTTCTTCCACCACTGATT	Gene expression
q*CK2*α F	GATCAGTTGGTGAGGATAGCCAAGG	Gene expression
q*CK2*α R	GGAGTGTCTGCCCAAGATATCGTTG	Gene expression
q*CK2*α′ F	GACCAGCTTGTTCGAATTGCCA	Gene expression
q*CK2*α′ R	CCAGCGCTTCCGTGAATGTTG	Gene expression
q*CK2*β F	ATGGACGTGTACACACCCAAGTC	Gene expression
q*CK2*β R	ACCATAGAGCCTGGGTACAAACTG	Gene expression
q*BAF155* F	GACTGGTGGTGCAGCTTCTACAGT	Gene expression
q*BAF155* R	GATCCATTCGGGATGGGTTCTGAA	Gene expression
q*BAF170* F	TTGCAGCTGCCTACAAATTCAA	Gene expression
q*BAF170* R	ACAGGATACACAACATGGGAGG	Gene expression
*Pax7* A ChIP F	GTGGCGACAAGGAAGTTCAAACAAAC	ChIP
*Pax7* A ChIP R	AAAGAAAGCCACTCCGCAACCTCTG	ChIP
S613A	GACCAGCCAGATGGCCGACCTCCCGGTG	SA-Brg1 mutation
S695A/S699A	CCAGATCCAGACGCCGATGACGTCGCTGAGGTGGACG	SA-Brg1 mutation
S1380A/S1382A	AAGGAGGTGGACTACGCCGACGCACTGACGGAGAAGC	SA-Brg1 mutation
S1570A	CGAGAAGGAGGATGACGCTGAAGGCGAGGAGGCT	SA-Brg1 mutation
S1575A	CAGTGAAGGCGAGGAGGCTGAGGAGGAGGAAGAG	SA-Brg1 mutation
S1586A	GCGAGGAGGAAGGCGCCGAATCCGAATCT	SA-Brg1 mutation
S1627A/S1631A	CCAAGCCGGTCGTGGCTGACGATGACGCTGAGGAGGAACAAG	SA-Brg1 mutation
S613E	GACCAGCCAGATGGAAGACCTCCCGGTG	SE-Brg1 mutation
S695E/S699E	CCAGATCCAGACGAAGATGACGTCGAAGAGGTGGACG	SE-Brg1 mutation
S1380E/S1382E	CACCGCAAGGAGGTGGACTACGAGGACGAGCTGACGGAGAAGCAGTGGCTC	SE-Brg1 mutation
S1570E	AATCGAGAAGGAGGATGACGAGGAAGGCGAGGAG	SE-Brg1 mutation
S1575E	GAAGGCGAGGAGGAGGAGGAGGAGGAAGAGGG	SE-Brg1 mutation
S1586E	GGGCGAGGAGGAAGGCGAGGAATCCGAATCTCGGT	SE-Brg1 mutation
S1627E/S1631E	CGAGCCAAGCCGGTCGTCGAGGACGATGACGAGGAGGAGGAACAAGAGGAG	SE-Brg1 mutation

### Primary cell culture

Mice were housed in the animal care facility at the University of Massachusetts Medical School (Worcester, MA) in accordance with the Institutional Animal Care and Use Committee guidelines. Mouse satellite cells were purified from whole-leg muscle from 3- to 6-week-old male and female wild-type C57Bl/6 or Brg1 conditional mice (41) by differential plating following Percoll sedimentation as described previously (73). Muscle tissue was excised, rinsed with Hanks' balanced salt solution (HBSS; Thermo Fisher Scientific), sliced into small pieces, and incubated with 0.1% Pronase in HBSS at 37 °C for 60 min. The cell suspension was filtered through a 100-μm cell sieve and resuspended in 3 ml of growth media (GM) containing a 1:1 mix of DMEM and F-10 media, 20% fetal bovine serum, 2% chick embryo extract (Sera Laboratories), and 25 ng/ml recombinant basic FGF (Millipore). Cells were filtered using a 40-μm cell sieve and centrifuged at 1,000 × *g* for 1 min at room temperature. The cells were placed in the top of a Percoll gradient consisting of 35% Percoll layered on top of 70% Percoll and centrifuged 20 min at 1,850 × *g* at room temperature. The myoblasts were obtained from the interface of the 70% Percoll fraction. Cells were washed with HBSS, centrifuged 5 min at 1,000 × *g*, and resuspended and plated in GM. Isolated myoblasts were grown on plates coated with 0.02% collagen (Advanced BioMatrix). Primary myoblasts were plated at 4 × 10^4^ cells/cm^2^ in GM for further analysis. Where indicated, primary myoblasts obtained from C57Bl/6 mice were incubated with increasing concentrations (1–10 μm) of TBB.

### Virus production and primary myoblast transduction

WT-, SA-, SE-Brg1 retroviruses were produced as described previously (74). Briefly, 10 μg of DNA for each construct were transfected into Bosc23 cells (75) using Lipofectamine 2000 (Thermo Fisher Scientific). The viral supernatant was collected after 48 h and filtered through a 0.45-μm syringe filter (Millipore). Primary myoblasts were infected with retrovirus in the presence of 8 μg/ml Polybrene and selected for 2 days with 1 μg/ml puromycin (Thermo Fisher Scientific). Surviving cells were then plated in GM at 4 × 10^4^ cells/cm^2^. Adenovirus expressing Cre-recombinase (Ad5CMVCre; Ad-Cre) or containing the empty vector (Ad5CMVempty; negative control) were used at a multiplicity of infection of 50 and were obtained from University of Iowa Gene Transfer Vector Core. After 48 h, transduced cells were re-plated at a density of 4 × 10^4^ cells/cm^2^ in normal GM, and Brg1 expression was evaluated for 4 days to determine the times when expression of the exogenous WT-, SA-, and SE-Brg1 mutants could be observed. Day three was chosen for further experimentation, consistent with prior work showing this was the time point at which the endogenous Brg1 was deleted (15).

### Primary myoblast proliferation assays

Primary myoblasts derived from satellite cells from the Brg1 c/c mutant mouse were transduced as indicated above to induce the expression of the WT-, SA-, and the SE-Brg1 mutants. Three days later, the cells were plated at a density of 1 × 10^4^ cells/cm^2^ in normal GM. Where indicated, wild-type C57Bl/6 were cultured with increasing concentrations of (1–10 μm) TBB (Cayman Chemicals). Samples were trypsinized at 24, 48, 72, and 96 h and counted in a Cell-O-Meter (Nexcelom). Student's *t* tests (two-tailed) were performed using Graphpad Prism Version 7 software.

### Protein expression and purification

WT-, SA-, and SE-Brg1 mutant proteins were expressed *in vitro* using TNT® Quick Coupled Transcription/Translation Systems (Promega) following the manufacturer's instructions. Briefly, 1 μg of DNA was incubated with 40 μl of TNT® master mix and 1 μl of 1 mm unlabeled methionine for 90 min at 30 °C. The resulting Brg1 proteins were immunoprecipitated using a polyclonal rabbit antisera against Brg1 (40) and PureProteome^TM^ protein A/G mix magnetic beads (Millipore). Samples were eluted in buffer containing 50 mm Tris-HCl, pH 7.5, 150 mm NaCl, 1% Nonidet P-40, 0.5% sodium deoxycholate, and Complete Protease Inhibitor Mixture (Roche Diagnostics). *In vitro* translated WT-, SA-, and SE-Brg1 proteins were analyzed by Western blotting using the polyclonal rabbit antisera against Brg1 (40). GST-Brg1 fragments (9) were produced in BL-21 cells following the autoinducing media protocol (76). For protein expression, cells were grown overnight at 37 °C in 2-YT medium supplemented with 100 μg/ml ampicillin. Bacterial pellets were sonicated; cell debris was removed by centrifugation, and the soluble fraction was incubated in a rotating mixer for 1 h at 4 °C with GST-agarose beads (Pierce) supplemented with Complete Protease Inhibitors. Samples were washed with PBS and eluted with 20 mm reduced glutathione (Sigma) in 50 mm Tris, pH 8.1. Expression of the GST-Brg1 proteins was verified by Coomassie Brilliant Blue Staining.

### Phosphorylation assays

Forty micrograms of purified WT-, SA-, SE-Brg1 and GST-Brg1 truncated proteins were incubated for 30 min at 30 °C with 50 units of CK2 (New England Biolabs) in 1× CK2 Reaction Buffer (20 mm Tris-HCl, 50 mm KCl, 10 mm MgCl_2_, pH 7.5) and supplemented with 200 μm ATP and γ-labeled ATP (PerkinElmer Life Sciences) to a final specific activity of 100 μCi/μmol. Where noted, TBB was added at the indicated concentrations. Then the reactions were separated by SDS-PAGE, transferred to PVDF membrane, exposed to a phosphorimager plate, and scanned using a Typhoon laser imager (Molecular Dynamics). Three (Fig. 1) or four (Fig. 4) independent experiments were performed and analyzed. Phosphorimages were quantified using ImageJ software, and the phosphorimage signal was normalized to either Brg1 or GST signal obtained from a parallel Western blotting.

### Antibodies

The antibodies used included the polyclonal rabbit antisera against Brg1 that was previously described (40). The rabbit anti-casein kinase IIα (H-286), mouse anti-casein kinase IIα′ (D-7), mouse anti-casein kinase IIβ (6D5), goat anti-Pax-3/7 antibody (N-19), rabbit anti-caspase-3 antibody (H-277), rabbit anti-Baf155 antibody (H-76), rabbit anti-Baf170 antibody (H-116), rabbit anti-lamin β1 (H-90), rabbit anti-pol II (N-20), and rabbit anti-MyoD (C-20) were purchased from Santa Cruz Biotechnology, Inc. Rabbit anti-PI3K p85 antibody, N-SH2 domain (ABS233) was obtained from Millipore Corp. Mouse anti-GAPDH-peroxidase-conjugated (G9295) was obtained from Sigma. Anti-myosin heavy chain (MF20, deposited by D. A. Fischman) antibody was obtained as a hybridoma supernatant from the Developmental Studies Hybridoma Bank (University of Iowa). Donkey anti-rabbit Alexa-488 (A21206) fluorescent secondary antibody was purchased from Thermo Fisher Scientific. Secondary antibodies anti-mouse and anti-rabbit HRP-coupled secondary antibodies were obtained from Pierce.

### Western blot analysis

Proliferating primary myoblasts were washed with PBS and solubilized with RIPA buffer (10 mm PIPES, pH 7.4, 150 mm NaCl, 2 mm EDTA, 1% Triton X-100, 0.5% sodium deoxycholate, and 10% glycerol) containing Complete Protease Inhibitor Mixture. Forty micrograms of each sample were prepared for SDS-PAGE by boiling in Laemmli buffer. The resolved proteins were electrotransferred to a PVDF membrane (Bio-Rad). The proteins of interest were detected with the specific polyclonal or monoclonal antibodies indicated, followed by species-appropriate peroxidase-conjugated antibodies (Pierce) and chemiluminescent detection (ECL PLUS; GE Healthcare). All Western blotting experiments were performed using samples from three independent experiments.

### Immunocytochemistry

Cells were grown on 12-well plates and differentiated by serum depletion (2% horse serum) for 48 h. Cells were fixed with PBS containing 10% formalin and then incubated overnight with anti-myosin heavy chain (MF20) antibody (1:100 dilution) along with 5% horse serum, 0.2% Triton X-100 in PBS. Secondary antibody binding and HRP staining were performed with the ABC universal kit and peroxidase substrate kit Vector VIP, respectively (Vector Laboratories), according to the manufacturer's protocol.

### Primary myoblast FRAP analysis

Cells were grown on glass bottom CELLview^TM^ Advanced TC culture dishes (Grenier Bio One) for 72 h in growth media. FRAP was performed with a Leica TCS SP5 II AOBS confocal laser-scanning microscope (Leica) using a ×63 1.4 n.a. oil immersion objective. FRAP images of the nucleus of live cells were scanned before, during, and after photobleaching at 2-s intervals at 515 nm. To photobleach YFP, maximal laser power was applied to a region of interest for 1 s. Leica Confocal software (LAS AF Lite) was used to measure the intensity of fluorescence in the bleached area, a non-bleached area, and the whole nucleus for the whole stack of images. The relative fluorescence intensity in the bleach zone was calculated according to Quaresma *et al.* (77) with normalization setting the pre-bleach intensity to 1 and the initial post-bleach intensity to zero. In this way, a true immobile fraction representing tightly bound complexes not exchanging over the time course of the experiment could be calculated. Data were analyzed and plotted using Graphpad Prism Version 7. Individual time points are presented as means with error bars showing standard errors of the mean. Half-times of recovery and immobile fractions were calculated by a best fit of each recovery data set to the equation *F*(*t*) = *F*_max_ (1 − *e*^−^*^kt^*).

### Immunoprecipitation

Cells were washed three times with ice-cold PBS and resuspended in lysis buffer (50 mm Tris-HCl, pH 7.5, 150 mm NaCl, 1% Nonidet P-40, 0.5% sodium deoxycholate, and Complete Protease Inhibitor Mixture). 250 μg of cell extract were incubated in a rotating mixer for 2 h with the primary antibody at 4 °C, followed by an overnight incubation with PureProteome^TM^ Protein A/G mix magnetic beads (Millipore). Samples were washed as indicated by the manufacturer, and immunoprecipitated proteins were eluted with freshly prepared IP buffer (10% glycerol, 50 mm Tris-HCl, pH 6.8, and 1 m NaCl) by incubating at room temperature for 1 h on a rotating mixer. Samples were analyzed by SDS-PAGE and Western blotting.

### Gene expression analysis

RNA was purified from three independent biological replicates of proliferating primary myoblasts with TRIzol (Thermo Fisher Scientific). cDNA synthesis was performed with 1 μg of RNA as template, random primers, and SuperScript III reverse transcriptase (Thermo Fisher Scientific) following the manufacturer's protocol. Quantitative RT-PCR was performed with Fast SYBR Green master mix (Thermo Fisher Scientific) on the ABI StepOne Plus Sequence Detection System (Applied Biosystems) using the primers listed in Table 1 and normalized to the levels of EF1-α expression. Two-tailed *t* tests were performed for statistical analyses using Graphpad Prism Version 7.

### Chromatin immunoprecipitation assays

ChIP assays using cultured mouse primary myoblasts were performed in triplicate from independent biological samples as described previously (15, 78, 79). Lysates from proliferating primary myoblasts were incubated overnight with polyclonal rabbit antisera against Brg1 (40), rabbit anti-Baf155 (H-76), rabbit anti-Baf170 (H-116), or normal rabbit IgG (Santa Cruz Biotechnology). Cross-linking was reversed, and DNA was purified using ChIP DNA Clean and Concentrator Columns (Zymo Research). Quantitative PCR was performed using Fast SYBR Green master mix on the ABI StepOne Plus Sequence Detection System. Primers used are listed in Table 1. Quantification was done using the comparative *Ct* method to obtain the percent of total input DNA pulled down by each antibody (80). Student's *t* tests (two-tailed) were performed using Graphpad Prism Version 7.

### Restriction enzyme accessibility assay

Nuclei from three independent biological replicates of proliferating primary myoblasts derived from mouse satellite cells were isolated by sucrose gradients. Briefly, the cells were trypsinized, resuspended in buffer containing 10 mm HEPES, pH 7.9, 10 mm KCl, 1.5 mm MgCl_2_, 0.5 mm DTT and homogenized with a Dounce tissue homogenizer. Nuclei were separated from cytoplasm by centrifuging the samples 5 min at 218 × *g* at 4 °C, resuspended in 3 ml of Buffer S1 (0.25 m sucrose, 10 mm MgCl_2_), then layered over 3 ml of Buffer S2 (0.35 m sucrose, 10 mm MgCl_2_), and centrifuged 5 min at 1,430 × *g* at 4 °C. Restriction enzyme accessibility assays were performed as described previously (81) using primers described in Table 1. Student's *t* tests (two-tailed) were performed using Graphpad Prism Version 7.

### Nuclear fractionation

Nuclear fractionation was performed following the high-salt isolation protocol previously described (48, 52). Primary myoblasts were washed with ice-cold PBS and extracted in cytoskeleton buffer (CSK: 10 mm PIPES, pH 6.8, 100 mm NaCl, 300 mm sucrose, 3 mm MgCl_2_, 1 mm EGTA, 1 mm DTT, and 0.5% (v/v) Triton X-100 and Complete Protease Inhibitor Mixture). The cytoskeletal fraction was separated from soluble proteins by centrifugation at 5,000 × *g* for 3 min. Chromatin was solubilized by DNase I digestion (1 unit, New England Biolabs) in CSK buffer plus protease inhibitors for 15 min at 37 °C. Then 0.25 m (NH_4_)_2_SO_4_ was added, and the samples were incubated for 5 min at 4 °C and centrifuged at 5,000 × *g* for 3 min. The pellet was washed with 2 m NaCl in CSK buffer for 5 min at 4 °C and then centrifuged. The remaining pellet containing the nuclear matrix was solubilized in 8 m urea buffer. Fractions were analyzed by SDS-PAGE and Western blotting.

## Author contributions

T. P.-B., B. T. N., and A. N. I. designed the experiments. T. P.-B., B. T. N., A. L. P., D. T. H., and J. M. S. performed the experiments. T. P.-B., B. T. N., A. L. P., D. T. H., J. A. N., and A. N. I. interpreted the data. T. P.-B., J. A. N., and A. N. I. wrote the manuscript with assistance and review by the other coauthors.

## Footnotes

2 The abbreviations used are:

CK2: casein kinase 2
TBB: 4,5,6,7-tetrabromobenzotriazole
Baf: BRG1- and Brahma-associated factors
MHC II: myosin heavy chain II
REAA: restriction enzyme accessibility assay
FRAP: fluorescence recovery after photobleaching
ESC: embryonic stem cell
GM: growth medium
RNA pol II: RNA polymerase II
HBSS: Hanks' balanced salt solution.
